# Polyarteritis nodosa with abdominal pain as the main symptoms: A case report

**DOI:** 10.1097/MD.0000000000046403

**Published:** 2025-12-12

**Authors:** Jin-long Chen, Xin-xin Fang

**Affiliations:** a Department of Gastroenterology, Guankou Township Center Hospital, Fujian, China; b Department of Gastroenterology, the Fuyang Affiliated Hospital of Anhui Medical University, Fuyang, Anhui Province, China.

**Keywords:** abdominal pain, case report, diagnosis, polyarteritis nodosa, treatment

## Abstract

**Rationale:**

Polyarteritis nodosa (PAN) is an uncommon systemic necrotizing vasculitis that is often misdiagnosed due to its rarity. Early diagnosis of PAN is critical, as prompt initiation of pharmacotherapy significantly improves the 5-year survival rate. However, a review of recent case reports indexed in PubMed reveals that the majority describe delayed PAN diagnosis, frequently associated with severe adverse outcomes, including fatalities. This pattern underscores the persistent diagnostic challenges in imaging for PAN. Thus, this case report contributes valuable insights to advance our understanding and recognition of PAN diagnosis.

**Patient concerns:**

A 65-year-old female patient presented with abdominal pain without obvious cause. Laboratory investigations revealed hypoalbuminemia, elevated D-dimer and fibrin degradation products, decreased IgG, IgM, and C3 levels, and a positive antinuclear antibody at a titer of 1:100 with a coarse speckled pattern. Nerve conduction electromyography and abdominal computer tomography angiography were performed. Based on the collective findings, PAN was ultimately diagnosed.

**Diagnoses:**

Based on the findings of weight loss ≥ 4 kg, leg tenderness, polyneuropathy, and abnormal arteriography, the patient was ultimately diagnosed with PAN.

**Interventions and outcomes:**

The patient received pulse methylprednisolone (500 mg/d × 3 days) and cyclophosphamide (400 mg q2w), followed by prednisone taper. Remission maintenance used prednisone 10 mg/d and methotrexate 7.5 mg/wk. After achieving remission, the patient maintained glucocorticoids and non-glucocorticoids for 2 years as consolidation therapy. Subsequently, all immunosuppressive agents were self-discontinued. At the telephone follow-up conducted 2 years after treatment cessation, the patient reported being asymptomatic and demonstrated no signs or symptoms related to PAN.

**Lessons:**

This case highlights the diagnostic challenges and complexities of maintaining long-term remission in PAN patients. Thus, it is crucial for gastroenterologists to maintain a high index of suspicion and include PAN in their differential diagnosis when evaluating patients.

## 1. Introduction

Polyarteritis nodosa (PAN), initially identified by Kussmaul A and Maier R in 1866, is a systemic necrotizing vasculitis. A French study reported a prevalence of 30.7 per million adults.^[1]^ This condition primarily targets medium-sized arteries, but small vessels can also be involved. Typically diagnosed in middle-aged and older adults, PAN affects men more than women. Unlike other vasculatures, PAN generally lacks antineutrophil cytoplasmic antibodies.^[2]^ Symptoms frequently include ulcers on fingers, ischemia, and painful skin nodules, with rare extra-articular manifestations and occasional abdominal pain. Most PAN cases are idiopathic, though often linked to neoplastic diseases, genetic mutations, and infections.^[3,4]^

This report discusses an elderly woman with abdominal pain. Reviewing the literature enhances the understanding of PAN’s history, imaging characteristics, and diagnostic approaches, providing a future clinical reference.

## 2. Case description

**Figure 1.**
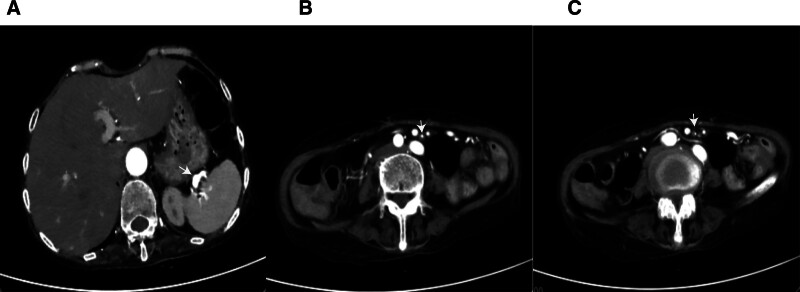
Pretreatment abdominal computer tomography angiography. A: Aneurysmal dilation in the distal splenic artery; B: Soft plaques at the origin of the superior mesenteric artery with mild luminal stenosis; C: Hypodensity in the middle and distal superior mesenteric artery and its branches, indicating emboli with moderate to severe luminal stenosis.

**Figure 2.**
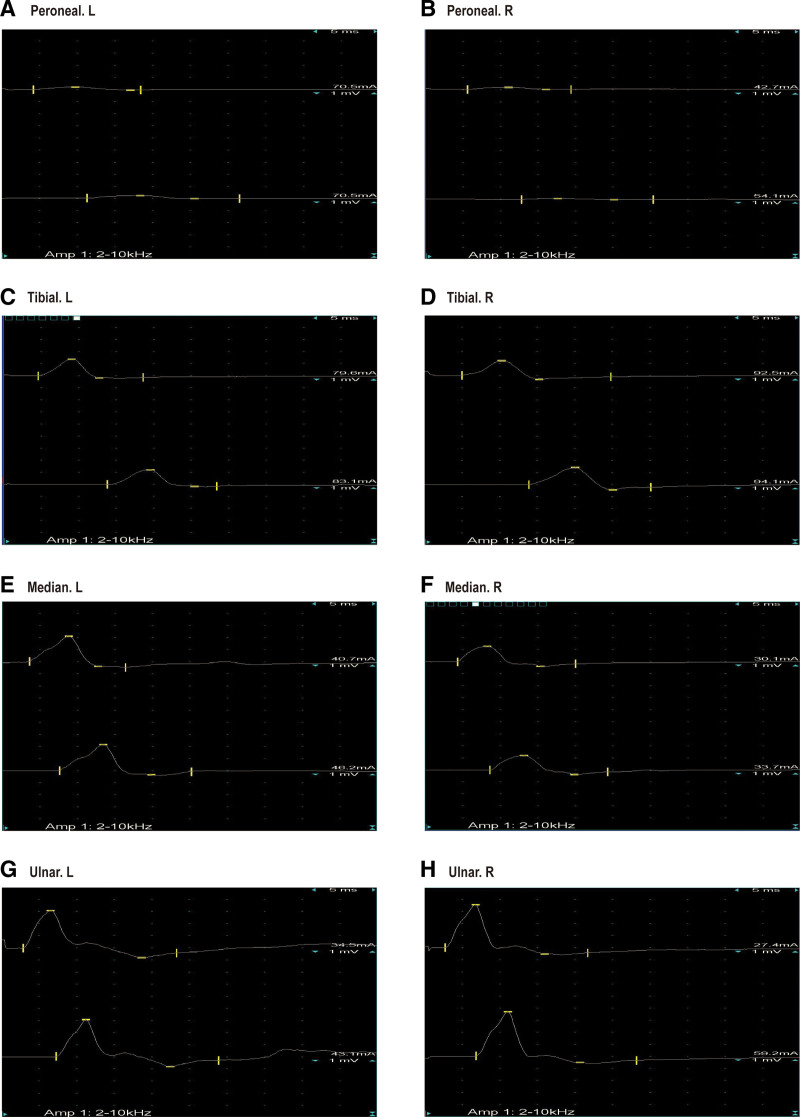
Electrical conduction in motor nerve examination in electromyography. A: Peroneal.L; B: Peroneal.R; C: Tibial.L; D: Tibial.R; E: Median.L; F: Median.R; G: Ulnar.L; H: Ulnar.R.

**Figure 3.**
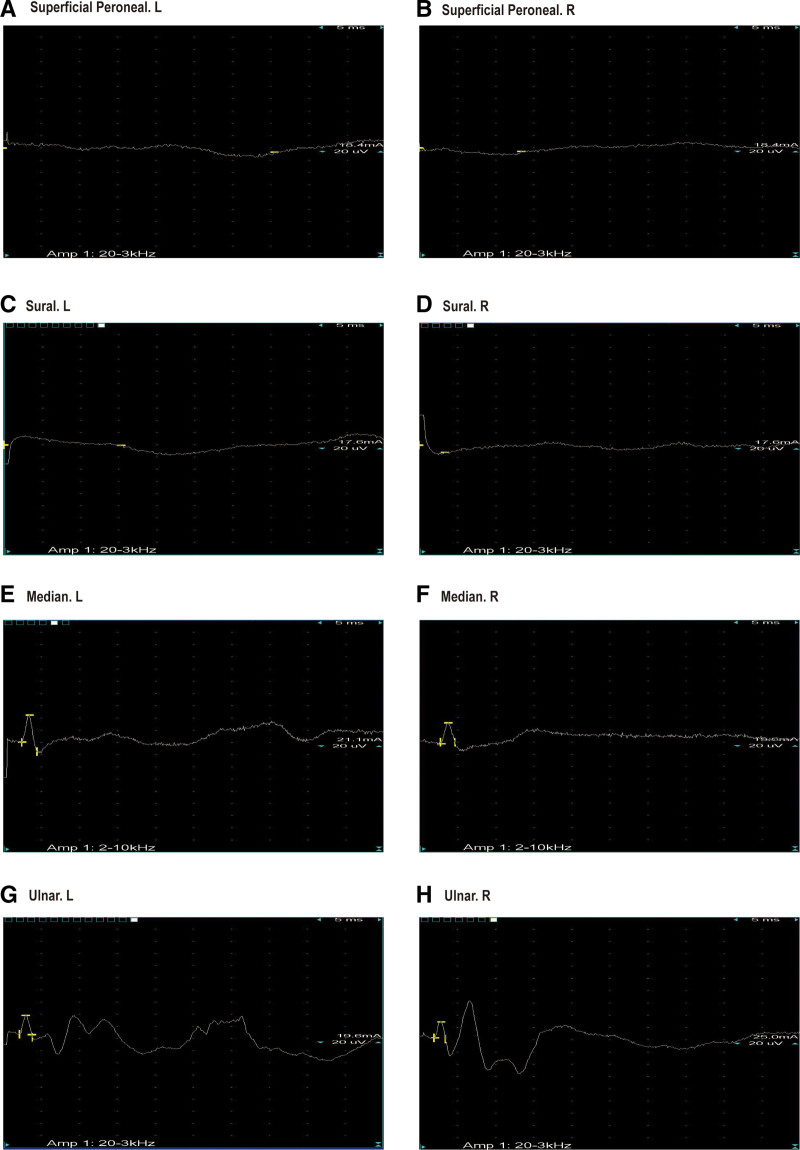
Electrical conduction in sensory nerve examination in electromyography. A: Superficial Peroneal.L; B: Superficial Peroneal.R; C: Sural.L; D: Sural.R; E: Median.L; F: Median.R; G: Ulnar.L; H: Ulnar.R.

**Figure 4.**
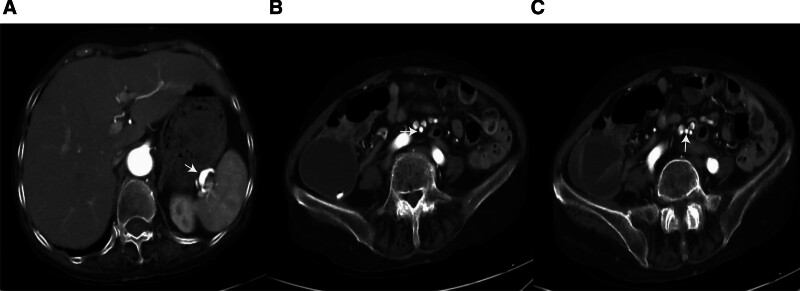
Abdominal computer tomography angiography after treatment. A: Persistent aneurysmal dilation in the distal splenic artery after treatment; B: There were no obvious abnormalities in the origin of the superior mesenteric artery; C: There were no obvious abnormalities in the middle and distal superior mesenteric artery and its branches.

**Figure 5.**
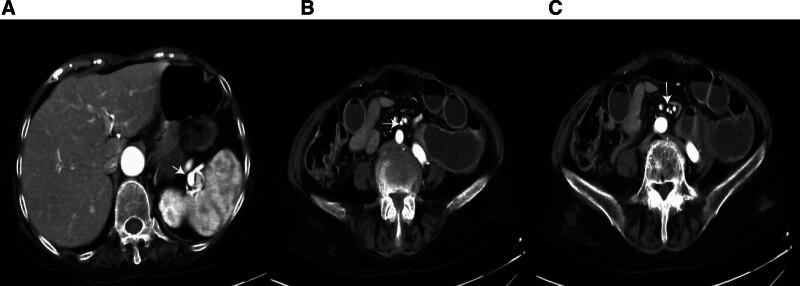
Abdominal computer tomography angiography after maintenance immunosuppressive therapy. A: Persistent aneurysmal dilation in the distal splenic artery after maintenance immunosuppressive treatment; B: Soft plaques at the origin of the superior mesenteric artery with mild luminal stenosis; C: There were no obvious abnormalities in the middle and distal superior mesenteric artery and its branches.

A 65-year-old woman presented on December 5, 2020, with a 5-month history of periumbilical abdominal pain. Her symptoms began on July 14, 2020, with severe intermittent periumbilical cramping and frequent yellow mucoid diarrhea (approximately 8 times daily). Initial hospitalization included treatment for septic shock with meropenem (1 g every 8 hours) stabilizing her vital signs, but abdominal pain and diarrhea persisted. Transferred to another hospital on August 4, 2020, abdominal computer tomography angiography (CTA) revealed key findings: aneurysmal dilation of the distal splenic artery (Fig. 1A), soft plaques with mild luminal stenosis at the superior mesenteric artery (SMA) origin (Fig. 1B), emboli causing moderate to severe stenosis in the mid-to-distal SMA and branches (Fig. 1C), and atherosclerotic plaques in the abdominal aorta and left common iliac artery. Suspected mesenteric embolism prompted anticoagulation, which was discontinued due to complications including hypotension, chest tightness, and abdominal ecchymoses, with subsequent improvement in coagulation. Following discharge on September 12, 2020, her stool normalized, but she experienced persistent nocturnal periumbilical colic (5–10 minute episodes) unresponsive to symptomatic treatment. She reported an unintentional 5 kg weight loss prior to admission. During hospitalization, the patient experienced episodes of paroxysmal peri-umbilical colic lasting about 5 to 10 minutes, accompanied by irregular and severe pain at the limb extremities and a sensation of heaviness in the feet and toes upon touch. A physical examination revealed the patient weighed 41 kg, appeared emaciated, exhibited peri-umbilical tenderness, and had severe pitting edema in the lower extremities. Laboratory investigations showed hypoalbuminemia, elevated D-dimer and fibrin degradation products, decreased IgG, IgM, and C3, and a positive antinuclear antibody at 1:100 coarse speckled pattern. Renal function and cardiac markers were normal; extensive serology (including hepatitis, ANCA, and comprehensive autoantibody panels) was negative. Imaging confirmed systemic arteriosclerosis via ultrasound (carotid, vertebral, and lower limbs) and electrocardiogram showed sinus rhythm. Neuromyography demonstrated severe motor (Fig. 2) and sensory (Fig. 3) axonal polyneuropathy, worse in the lower extremities (Table 1). She met the 1990 American College of Rheumatology criteria for PAN based on weight loss ≥ 4 kg, leg tenderness, polyneuropathy, and arteriographic abnormalities. The 1996 Five-Factor Score (FFS, 1996) included the following parameters that were associated with higher risk of death: proteinuria > 1 g/d, renal insufficiency (stabilized peak creatinine 140 µmol/L), cardiomyopathy, severe gastrointestinal manifestations, and central nervous system involvement. Her FFS (1996) score was 2 due to gastrointestinal and neurological involvement. Treatment comprised pulse methylprednisolone (500 mg/d for 3 days) followed by a prednisone taper and cyclophosphamide (400 mg every 2 weeks). By 6 months, her symptoms resolved completely, and follow-up CTA showed normalization of the SMA (Fig. 4A–C). Maintenance therapy with prednisone (10 mg/d) and methotrexate (7.5 mg/wk) was initiated. After the remission treatment, she continued with consolidation treatment for 2 years. Repeat CTA then revealed mixed plaques with mild stenosis at the SMA origin (Fig. 5A–C). As of the last phone follow-up on June 13, 2025, the patient remains alive and asymptomatic with no clinical signs or symptoms attributable to active PAN.

**Table 1:** Electromyography results.

	Left			Right		
	Latent period (ms)	Amplitude (μV)	Speed (m/s)	Latent period (ms)	Amplitude (μV)	Speed (m/s)
Motor nerve conduction						
Peroneal						
Ankle	4.2	300.0	–	5.6	200.0	–
Fibula (head)	11.3	300.0	44.0	12.8	200.0	43.0
Tibial						
Ankle	4.6	1600.0	–	5.0	1600.0	–
Popliteal fossa	13.8	1400.0	40.0	13.9	1800.0	41.0
Median						
Wrist	3.6	2500.0	–	4.3	1700.0	–
Below elbow	7.6	2500.0	54.0	8.6	1600.0	49.0
Ulnar						
Wrist	2.9	3900.0	–	2.7	4100.0	–
Below elbow	7.3	3900.0	52.0	6.8	4200.0	54.0
Sensory nerve conduction						
Superficial peroneal						
Mid calf	Not led out	Not led out	Not led out	Not led out	Not led out	Not led out
Sural						
Lower leg	Not led out	Not led out	Not led out	Not led out	Not led out	Not led out
Median						
Wrist	2.4	44.0	49.0	2.7	36.0	43.0
Ulnar						
Wrist	2.1	31.0	48.0	1.9	26.0	53.0

## 3. Discussion

This article presents a case report of an elderly woman presenting with primary symptoms of abdominal pain. Imaging studies identified superior mesenteric artery stenosis and splenic artery aneurysms. Clinical and imaging findings led to a diagnosis of PAN with an FFS (1996) score of 2. Combining glucocorticoids (GCs) and immunosuppressants ameliorated her symptoms. The understanding of PAN has evolved, marked by significant milestones in 1842, 1866, and 1970. However, the etiology remains largely undisclosed, with high rates of missed and misdiagnoses, and the selection of treatment protocols post-remission still necessitates further discussion and research.^[5,6]^ The pathogenesis of PAN is unclear, likely involving infections and genetics. Research suggests links between PAN and viral infections (hepatitis B virus, hepatitis C virus, human immunodeficiency virus) and streptococcal infections. Patients with PAN related to hepatitis B virus can achieve remission with treatment.^[7]^

In this case, despite comprehensive etiological testing early in the disease course, no specific pathogenic bacteria were identified. Given the potential hereditary component, it is notable that both parents had a history of suspected rheumatic diseases. However, with both parents deceased and limited close relatives, genetic testing to determine if the patient carries genetic predispositions to PAN is not feasible. The widely accepted PAN diagnostic criteria were established by the American College of Rheumatology in 1990. Further consideration is warranted on how to reduce the high rates of missed diagnoses and to develop standardized and rational methods for monitoring the disease. In this case, although the initial abdominal CTA revealed distal splenic artery aneurysmal dilatation-a radiological hallmark of PAN-no other diagnostic evidence was present, which may have contributed to the delayed diagnosis. Treatment of newly diagnosed non-severe active PAN typically involves GCs and immunosuppressants, adjusted according to organ involvement and disease severity. Glucocorticoids alone may be sufficient for mild cases, while cyclophosphamide is advised for those with an FFS score ≥ 1 to enhance survival.^[8–11]^ For patients who achieve remission, the guidelines suggest that treatment with non-GCs immunosuppressants should be discontinued after 18 months, while the duration of GCs treatment is determined by the patient’s assessment and preferences.

In this case, the patientunderwent a treatment regimen of methylprednisolone and cyclophosphamide during the active phase, addressing the involvement of the digestive and nervous systems. After achieving remission, the patient maintained GCs and non-GCs for 2 years as consolidation therapy. Nevertheless, a surveillance abdominal CTA demonstrated recurrent mesenteric vascular lesions. Notably, the patient remained clinically asymptomatic with no evidence of PAN disease activity. Subsequently, all immunosuppressive agents were self-discontinued. Throughout the following 2-year treatment-free period, the patient continued to exhibit no PAN-related symptoms and remained progression-free. Therefore, selecting appropriate maintenance therapy necessitates further high-quality research. The prognosis of PAN depends on various factors. An acute onset requires prompt, accurate diagnosis and aggressive treatment to achieve a favorable outcome. Research shows that patients with an FFS (1996) score of ≥ 2 have a 5-year mortality rate about 4 times higher than those with a score of 0.^[12]^ Although the diagnosis was delayed during the initial disease presentation, and a FFS (1996) of 2 indicated a poor prognosis, the patient has nevertheless survived for 4.5 years to date following immunosuppressive therapy. Despite recurrent subclinical vascular lesions on CTA after 2 years, the patient remained asymptomatic for 2+ years off therapy – suggesting prolonged immunosuppression may not be mandatory in clinically stable PAN.

There are some shortcomings in the medical records. Firstly, diagnosing PAN was delayed during initial hospital evaluation due to the patient’s primary symptoms of abdominal pain without peripheral vascular neuropathy, commonly seen in PAN. Secondly, while the patient met the 1990 diagnostic criteria for PAN by the American Rheumatology Association, the lack of definitive vascular pathology confirmation was a limitation in this case.

## 4. Conclusion

PAN is an uncommon, acute, and potentially fatal condition that often presents without distinctive clinical features, complicating its diagnosis. Autoantibody tests frequently return negative results, adding to the diagnostic challenge. Although symptoms such as abdominal pain and diarrhea are atypical for PAN, they may indicate gastrointestinal involvement, which is associated with a poorer prognosis. Thus, it is crucial for gastroenterologists to maintain a high index of suspicion and include PAN in their differential diagnosis when evaluating patients.

## Acknowledgments

We appreciate the guidance of Dr Yi Han from the Department of Gastroenterology, Fuyang Hospital of Anhui Medical University, Fuyang, Anhui, China, for diagnosis and treatment assistance.

## Author contributions

**Conceptualization:** Jin-long Chen, Xin-xin Fang.

**Data curation:** Jin-long Chen, Xin-xin Fang.

**Funding acquisition:** Xin-xin Fang.

**Project administration:** Jin-long Chen.

**Supervision:** Xin-xin Fang.

**Validation:** Xin-xin Fang.

**Writing – original draft:** Jin-long Chen.

**Writing – review & editing:** Xin-xin Fang.
